# A scoping review of gamified applications in English language teaching: a comparative discussion with medical education

**DOI:** 10.1186/s12909-025-06822-7

**Published:** 2025-02-20

**Authors:** Zahra Zolfaghari, Zahra Karimian, Nahid Zarifsanaiey, Amir Yousef Farahmandi

**Affiliations:** 1 Department of E-Learning in Medical Sciences, Virtual School, Center of Excellence in e-Learning, Student Research Committee, Shiraz University of Medical Sciences, Neshat Street, Sadra & Sina Hall, Shiraz, Iran; 2 Department of e- Learning in Medical Sciences, Virtual School and Center of Excellence in e-Learning, Shiraz University of Medical Sciences, Shiraz, Iran; 3 Department of e-Learning in Medical Sciences, Virtual School and Center of Excellence in e-Learning, Shiraz University of Medical Sciences, Shiraz, Iran; 4 Department of English Language, School of Paramedical Sciences, Shiraz University of Medical Sciences, Shiraz, Iran

**Keywords:** Gamification, Education, Medical, Language, TEFL, Technology, Game

## Abstract

**Background:**

With the increasing integration of technology in education, understanding how gamification can enhance language learning is crucial for developing effective teaching strategies. This scoping review explored the current landscape of gamified applications within TEFL while discussing comparative insights from medical education to outline effectiveness and potential across disciplines.

**Method:**

A systematic search following the PRISMA-ScR protocol was conducted across PubMed, Scopus, Web of Science, CINAHL, Cochrane Library, ERIC, and Embase databases to identify studies published between 2010 and 2024. English or Persian Quantitative, qualitative, or mixed-methods research examining interventional approaches or gamified applications in TEFL reporting on the effectiveness of these applications, detailing their design and implementation strategies, and identify the target student populations were included.

**Results:**

A total of 33 studies were included in the review, with an emphasis on their publication year, geographical distribution, study designs, delivery modes, technology utilization, gamification elements, and measurement tools. The review revealed that most publications were concentrated in 2018, with a significant number originating from Asia. The total number of students involved in the intervention studies was 2,531. Quasi-experimental pretest/posttest designs were the most common methodologies used, followed by mixed-method approaches. Online delivery emerged as the predominant mode of instruction, with mobile learning technologies frequently utilized. Feedback was identified as the most commonly employed gamification element, followed by challenges that fostered learner engagement.

**Conclusion:**

The findings highlighted the effectiveness of gamified applications in enhancing motivation and engagement among language learners. Immediate feedback and interactive elements are critical components that contribute to improved learning outcomes. As interest in gamification continues to grow, further research is warranted to explore its long-term impacts and potential applications across diverse educational contexts. This review underscores the importance of integrating gamification into TEFL to create dynamic and effective learning environments.

## Introduction

The rapid advancement of technology has transformed the education landscape, particularly in Teaching English as a foreign language (TEFL) [1]. The application of gamification in TEFL field instruction is one innovative approach that has gained significant attention in recent years [2]. This intersection of technology and education has opened new opportunities for interactive and immersive learning experiences [3]. Gamification involves integrating game-like elements, including points, badges, leaderboards, and challenges, into non-game contexts to enhance engagement, motivation, and learning outcomes [4]. Incorporating gamification into EFL instruction is particularly important as learners often perceive language learning as a daunting and monotonous experience [2]. As globalization continues to emphasize the importance of English proficiency, educators are increasingly seeking effective methods to engage learners and enhance their language skills [5]. By incorporating game-based elements, educators can create a more interactive, enjoyable, and rewarding learning environment, leading to increased student participation, improved language proficiency, and better retention of the target language [6].

Several studies have demonstrated the positive impact of gamification on student engagement in TEFL. Alrabab et al. (2023) explored the effects of gamification on academic achievement and motivation, found that incorporating gaming elements including interactive board games and role-playing scenarios significantly increased student participation and knowledge retention [5]. Similarly, Dehghanzadeha H et al. [7] highlighted the positive effects of gamification on learners’ learning experiences and outcomes, including being enjoyable, engaging, motivating, and fun. In a systematic review study investigating the effects of gamification on TEFL, the tendency of students to use games to learn English was analyzed. Their findings suggested that the gamification increase enjoyment, enhance student motivation, and improve participation while facilitating autonomous learning [8].

In another study, Darque Pinto et al. (2021) recognized the potential of using Virtual Reality(VR) technologies combined with gaming strategies to benefit and support TEFL. The study suggested that gamification in VR environments showed promise for improving second language acquisition and indicated that more work is needed to fully understand and optimize the use of VR and gamification in language learning [9]. A mixed-method study, analyzing the effects of gamification on increasing motivation and participation in TEFL, revealed that utilizing gamification in TEFL was meaningful and constructive [10].

The advantages of gamified learning go beyond simple engagement. Studies indicate that gamification can alleviate anxiety linked to language learning, thus fostering a more supportive environment for students to express themselves and practice their skills [11, 12]. Gamification encourages collaboration among students by motivating them to work together toward common goals, which is especially helpful for developing communication skills. Incorporating elements like points, badges, and leaderboards has proven to be an effective way to incentivize participation and foster a sense of achievement among learners [13].

However, a systematic review by Zhang et al. (2021) revealed contradictory findings regarding the impact of gamification on student motivation and learning outcomes, indicating that while some studies report positive effects, others suggest minimal or no long-term benefits [2]. Moreover, recent literature has emphasized the need for further research on how specific gamification elements influence educational outcomes across various contexts. Alahmari et al. (2023) noted that gamification methods may not be universally applicable across different educational settings and called for more targeted research to understand their effectiveness in diverse learning environments [14].

Despite the growing interest in gamified applications for TEFL instruction, and the valuable insights these studies provided into the trends and key findings related to gamification in TEFL, there is a need for a comprehensive understanding of the current state of research in this field via detailed information on the methodologies, instructional designs, limitations, specific game elements, and their effectiveness. This scoping review aimed to address the gap in research by examining the current landscape of gamified applications in English language teaching. It explores various aspects, including the geographical distribution of studies, the research designs employed, the technology used, and the specific gamification elements most frequently applied in language instruction. In addition to focusing on TEFL, this review draws comparative insights from medical education, where similar gamification strategies have been implemented to enhance student engagement and learning outcomes. This review seeks to provide a broader understanding of how gamification can effectively support learning across diverse educational contexts. Ultimately, the goal is to offer valuable insights for educators, curriculum developers, and instructional designers interested in using gamified approaches to enhance language learning. The findings may also inform future research directions and practical applications of gamification in both TEFL and other educational domains, highlighting its potential to create dynamic and effective learning environments.

### Objective and research questions

This study examined the existing evidence on using gamified applications as an educational approach for TEFL. The following research questions were addressed:

1. What types of gamified applications or techniques have been used to TEFL?
2. What methodologies and technological tools are most commonly employed in gamified applications within TEFL?
3. What are the reported outcomes and effectiveness of gamified applications for TEFL?
4. What challenges do educators face when implementing gamified applications in TEFL?

## Method

This scoping review was conducted to provide a broad, comprehensive mapping of the existing literature on gamification in TEFL. This allows the researchers to survey the current state of research in the field, identify areas where further study is needed, and uncover research gaps [15]. The present review was conducted following the PRISMA-ScR protocol. The review framework consisted of five steps: (1) defining the research question; (2) identifying relevant studies; (3) selecting the studies; (4) charting the data; and (5) collecting, summarizing, and reporting the results.

Identifying relevant studies, the authors devised the search strategy. The period covered was between 2010 and 2024. The reason for choosing this time frame is that around 2010, the concept of gamification became popular in educational settings, leading to a significant shift in teaching methods. This period witnessed a rise in research aimed at incorporating game elements into language learning practices [16].

The following indexing databases were searched: PubMed, Scopus, Web of Science, CINAHL, Cochrane Library, ERIC, and Embase. Table 1. Shows the detail of search terms used in databases.

**Table 1:** Search terms utilized in Pubmed, Scopus, web of sciences, and Eric Databases

Database	Search Term Used
PubMed	(gamification [Title/Abstract] OR “gamified applications“[Title/Abstract] OR “game applications“[Title/Abstract] OR “English language teaching“[Title/Abstract] OR “foreign language education“[Title/Abstract]) AND (education[Title/Abstract] OR learning[Title/Abstract] OR teaching[Title/Abstract] OR students[Title/Abstract] OR “educational settings“[Title/Abstract])
Scopus	TITLE-ABS-KEY(gamification OR “gamified applications” OR “game applications”) AND TITLE-ABS-KEY(“English language teaching” OR “foreign language education”) AND TITLE-ABS-KEY(education OR learning OR teaching OR students)
Web of Science	TS=(gamification OR “gamified applications” OR “game applications”) AND TS=(“English language teaching” OR “foreign language education”) AND TS=(education OR learning OR teaching)
Eric	(gamification OR “game-based learning”) AND (“English language teaching” OR “foreign language education”)

### Inclusion criteria

- The inclusion criteria were quantitative, qualitative, mixed-methods, interventional, and studies exploring gamified applications or gamification techniques in TEFL.
- Studies report on the outcomes and effectiveness of using gamified applications for TEFL.
- Studies that described the design features, implementation strategies, and target student populations of the gamified applications used in TEFL.
- Studies published in English or Persian.

Our decision to focus on studies that report on the outcomes and effectiveness of gamified applications in TEFL stems from the studies’ objective to map existing literature that demonstrates the tangible benefits of gamification. We aimed to identify research that not only describes gamification techniques but also evaluates their impact on learning outcomes. This focus allows us to highlight studies that contribute significantly to understanding how gamified applications influence language acquisition.

In this review, data were extracted from articles focusing on gamified applications in TEFL. The extracted data were charted for several key items to provide a comprehensive overview of the current landscape in this field. Specifically, we documented the following elements for each study: author, publication year, country of origin, publication source, sample size, target population, population age, duration of the study, design aims, type of gamification employed, outcomes measured, measurement tools used for outcomes, modes of delivery for gamification, benefits or effects related to gamification technology, and issues or needs associated with gamification technology.

The selection of these specific data items was guided by the objectives of our review and their relevance to our research questions. By extracting this information, we aimed to ensure that our analysis captures essential trends and insights regarding the implementation and effectiveness of gamified approaches in TEFL.

### Exclusion criteria

- Studies that only describe the development of gamified learning activities without reporting on their effects.
- Studies that focus only on qualitative data without quantitative outcomes.
- Studies not published in English or Persian.
- Studies that were not available in full text.
- Studies that do not specifically address gamification in TEFL.

## The PCC framework

### Population (participants)

The population of interest for this scoping review would be students learning English as a Foreign Language (EFL), including those in formal educational settings, language schools, and online learners.

#### Concept

The key concept is using gamified applications or techniques as an educational approach to TEFL.

#### Context

The context would be educational settings, such as language institutions, online platforms, or blended learning environments, where gamified applications are utilized for TEFL. (Table 2)

**Table 2:** Search terms of the study based on the PCC Framework

Patient, Concept, Context (PCC)	Target	Search words
Participants	Students	• Students • Learners • Online Learners • English language learners • Foreign language students
Concept	Gamification for TEFL	• Game application • Gamification • Game-based learning • Serious games • Educational games • Game elements • Gamified applications • Gamified platforms
Context	Educational setting	• Foreign language education • English as a Foreign Language (EFL) • Second language learning • Language instruction Language teaching • Vocabulary, reading, listening, speaking

### Quality assessment

In the initial screening phase, two independent reviewers meticulously evaluated each study included in the scoping review on gamification in TEFL. Each reviewer independently assessed the studies using predefined inclusion criteria, focusing on methodological rigor, relevance to the research questions, and alignment with the review’s objectives. When discrepancies emerged between the reviewers’ initial assessments, they engaged in comprehensive discussions to reconcile differences, ultimately reaching a consensus through collaborative dialogue. This consensus-based approach ensured a systematic and objective evaluation of the studies, minimizing potential individual biases and enhancing the reliability of the study selection process. The detailed appraisal involved a systematic scoring process using predefined criteria developed specifically for assessing studies on gamification in language education. Reviewers comprehensively documented their rationale for each scoring decision, creating a transparent and traceable assessment methodology. The scoring framework encompassed multiple dimensions, including research design quality, methodological rigor, statistical significance, and potential limitations.

### Collating, summarizing, and analyzing data

After quality assessment phase, data were categorized and analyzed. A table summarizing the articles’ characteristics and findings was prepared, and a list of articles was compiled. An overview of the studies was conducted by systematically counting the geographic distribution of the articles, year of publication, outcomes, and content analysis of the studies to identify the benefits, effects, and challenges related to gamification in TEFL. The results were shared among the researchers, and the classification and results of content analysis were discussed to ensure consensus on the perceptions. In cases where differences arose, we employed a consensus-building approach. Researchers engaged in constructive discussions to evaluate the evidence for each perspective. This process often involved revisiting the original articles to ensure that interpretations were grounded in the data. Content analysis was conducted using the conventional content analysis method by Hsieh and Shannon (2005) [17]. The article’s content was read and summarized in the first stage according to semantic units (primary codes). In the second stage, primary codes were grouped and converted into secondary codes in light of the research objectives and analysis categories. In the third step, the codes were grouped into subcategories by comparing them from the viewpoint of similarity and difference. As for the fourth step, the codes were categorized based on the relationships among the subcategories.

## Results

**Fig. 1:**
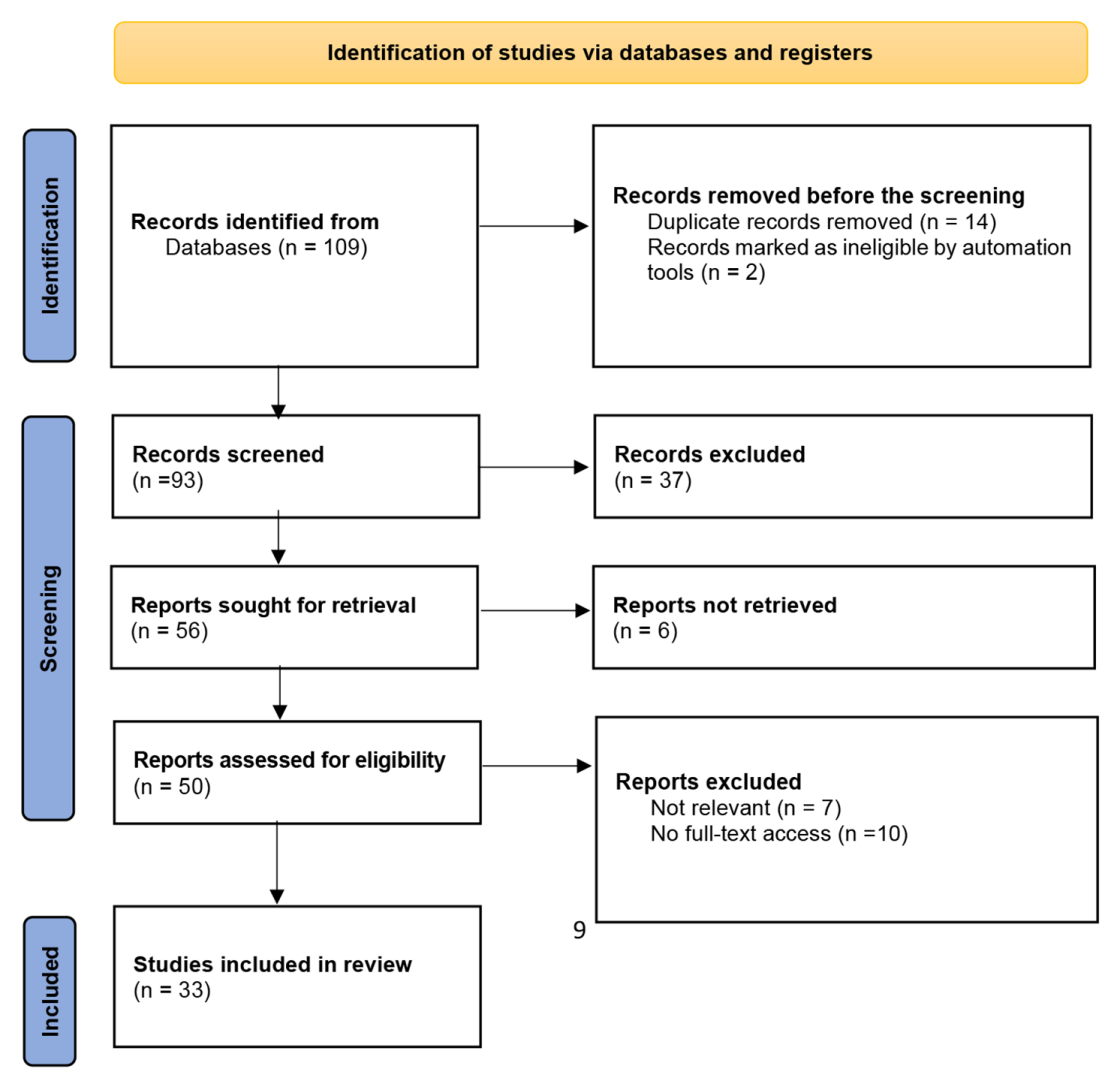
PRISMA flow diagram of the study

The primary searches based on the aim of this study yielded over 109 articles; after applying the inclusion and exclusion criteria, 33 articles met the criteria for review and analysis, Fig. 1 presents the PRISMA flow diagram of the study.

### Charting the data

Data were extracted from the 33 articles and were charted for the following items: author, publication year, country, publication source, sample size and target population, population age, and duration of the study (Table 3) design aims, type of gamification, outcomes, measurement tool of outcomes, gamification modes of delivery, benefits or effects related to gamification technology, and issues or needs related to gamification technology. The quality of the articles was not examined as this step was not part of the study’s objective.

**Table 3:** Characteristics of included articles on Gamified Applications in English Language Teaching from 2010 to 2024

No.	Authors	Publication year	Country	Publication source	Sample size	Target population	Population age	Intervention Duration
1	A. I. L. Burgos et al [18]	2024	Ecuador (South America)	Education Quarterly Reviews	*N* = 14	Students attending two different educational centers located in Manta-Ecuador	8–9 years	6 months
2	Shen Z et al [19]	2024	China	Frontiers in Psychology	*N* = 413	Learners enrolled in linguistic programs, learning English as a second language	18–45 years and above	Not-applicable
3	Huseinović L. [20]	2023	Bosnia and Herzegovina	MAP Education and Humanities	*N* = 202	Higher education students enrolled in public and private universities learning EFL	18–28 years	Not-applicable
4	EA Simbaña-Simbaña et al [21]	2023	Ecuador, South America	CIENCIAMATRIA	*N* = 50	Students from a Private school in El Quinche	13–14 years	12 sessions
5	Önal, Nezih et al [22]	2022	Turkey	Interactive Learning Environments	*N* = 110	Preparatory class students of a school of foreign languages in a state university	Not-applicable	8 weeks
6	Chan Sumie et al [23]	2022	China	SN COMPUT	*N* = 76	University and college students and teachers in Hong Kong in various English Language courses at different levels	17–22 years	Before and during the pandemic period
7	Xiuhan Li et al. [24]	2022	China	Sustainability	*N* = 217	L2 English learners	8–10 years	1 semester
8	Sorayyaei Azar et al [25]	2020	Malaysia	Universal Journal of Educational Research	*N* = 63	Management and Science University interns	22–28 years	Not-applicable
9	Marina Purgina et al [26]	2020	Japan	Journal of educational computing research	*N* = 21	Sophomore computer science students at a public Japanese university	12–22 years	Not-applicable
10	Wong Mee Mee et al [27]	2020	Malaysia	International Journal of Evaluation and Research in Education	*N* = 33	Pre-service teacher at local primary schools	Not-applicable	16 weeks
11	YANES N et al [28]	2019	Saudi Arabia	International Conference on Computer and Information Sciences (ICCIS)	*N* = 49	Students of Computer and Information Science College at Jouf University	Not-applicable	1 session
12	Lam, Hew & Chiu [29]	2018	China	Language, Learning and Technology	*N* = 72	Secondary girl’s school students	16–17 years	7 weeks
13	Ling [30]	2018	Singapore	Online Learning	*N* = 22	First-year undergraduates of the National University of Singapore (Most students were Singaporean, with a number coming from regional countries, such as Malaysia, Indonesia, Thailand, China, and India)	19–25 years	1 week
14	Castañeda, Guerra, & Ferro [31]	2018	Columbia	Interactive Technology and Smart Education.	*N* = 163	Elementary Students at the I.E.D. Técnico Industrial de Tocancipá,	5–8 years	1 session
15	Kirsi Korkealehto [32]	2018	Finland	International Journal on Media, Technology and Lifelong Learning	*N* = 23	First-year healthcare students at the University of Applied Sciences	19–51 years	10 weeks
16	Won and Kim [33]	2018	South Korea	IGI Global	*N* = 52	Learners of higher education who use Facebook	Adult	6 weeks
17	Sun and Hsieh [34]	2018	Taiwan	Journal of Educational Technology & Society	*N* = 144	English learners	7th grade	2 weeks(4sessions)
18	Homer R et al [35]	2018	China	Journal of Educational Technology & Society	*N* = 120	Elementary school ESL students across eight classes	1,2,3,4th grades	16 weeks
19	Guaqueta and colleuges [36]	2018	Spain	English Language Teaching	*N* = 20	EFL learners and school students in a rural school in Tolima	14–17 years	6 months
20	Bustillo, J, et al [37]	2017	Colombia	Sistemas & Telemática	*N* = 13	High school and adult learners in foreign language courses	19 years	2 month
21	Mchucha, Issa & Tibok [38]	2017	Malaysia	International Journal of Management and Applied Science (IJMAS)	*N* = 225	Undergraduate students from an English proficiency class at the University of Malaysia Sabah	Adults	25 min
22	Zhou, Yu & Shi (2017) [39]	2017	China	HCIBGO	*N* = 15	Language learners	Not-applicable	1 session
23	Medina and Hurtado[40]	2017	Ecuador (South America)	Revista Publicando	*N* = 70	Higher education students in an English language classroom, Universidad Técnica de Ambato	20–22 years	10 weeks
24	Girardelli, D. [41]	2017	China	Communication teacher	*N* = 24	Chinese EFL sophomore enrolled in a Sino-American international branch campus accredited by the Middle States Commission on Higher Education	Not-applicable	75 min
25	Baldauf et al [42]	2017	Austria (Europe)	Proceedings of the 16th International Conference on Mobile and Ubiquitous Multimedia	*N* = 62	Teachers, and parents/students in three classes learning EFL	13–14 years	5 weeks
26	Hung H.T [43]	2017	Taiwan	Interactive Learning Environments	*N* = 44	Students of English majors at a medium-sized university	20–22 years	2 weeks
27	Kayımbaşıoğlu et al. [44]	2016	Turkey	Procedia Computer Science	*N* = 60	Preschoolers	Not-applicable	5 years
28	Zarzycka-Piskorz [45]	2016	Poland	Teaching English with Technology	*N* = 112	Upper intermediate students of General English language course at the Pedagogical University, Cracow	19–24 years	3 weeks
29	Zhi Quan [46]	2016	China	Journal of Computers in Education	*N* = 28	Pre-university intermediate ESL (English as a second language)	Not-applicable	2 semesters
30	Hasegawa et al [47]	2015	Japan	SpringerPlus	*N* = 27	Undergraduates and graduate Learners of English vocabulary	19–24 years	1 week
31	Lui [48]	2014	China	(CELC) Symposium	*N* = 101	Five groups of students, from different faculties who had to take English I or Business English in the second semester of their first year of the undergraduate program	18–20 years	10 min
32	Abrams et al [49]	2014	United States	Journal of Adolescent & Adult Literacy	*N* = 31	Students from other countries	11th grade	One school year
33	Sandberg et al [50]	2011	Netherlands	Computers & Education	*N* = 75	Primary school students from three different schools	8–10 years	2 weeks

### Characteristics of the selected studies

**Fig. 2:**
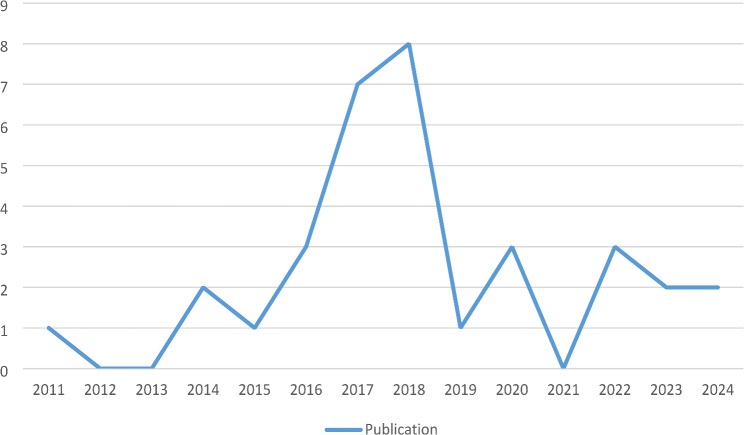
The trend of the publication year of reviewed studies

Among 33 studies reviewed in this study two studies were published in 2024 [18, 19], two were 2023 [20, 21], three were 2022 [22–24], three were 2020 [25–27], one was 2019 [28], eight were published in 2018 [29–36], seven were 2017 [37–43], three were published in 2016 [44–46], one was 2015 [47], two were 2014 [48, 49] and one 2011 [50](Fig. 2).

**Fig. 3:**
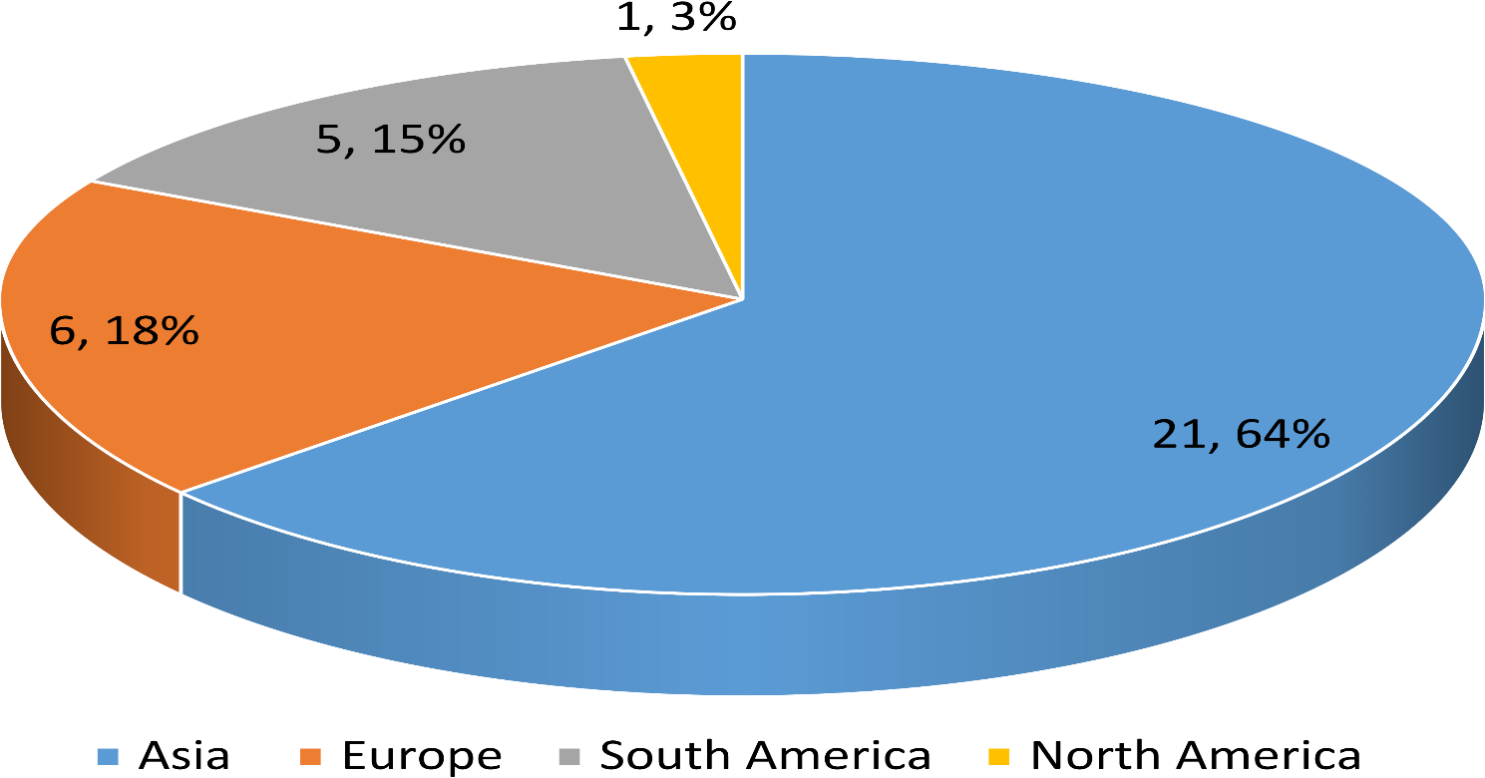
Data on the continents of publication

Data on the continents of publication are shown in Fig. 3. The most common continents of publication were Asia with 21 studies: China [19, 23, 24, 29, 35, 39, 41, 46, 48], Malaysia [25, 27, 38], Taiwan [34, 43], Turkey [22, 44], Japan [26, 47], Saudi Arabia [28], Singapore [30], and South Korea [33], six from Europe: Austria( [42], Poland [45], Bosnia and Herzegovina [20], Finland [32], Spain [36], and the Netherlands [50], five from South America: Ecuador(South America) [18, 21, 40], and Columbia [31, 37], and one from North America: United States [49].

The total number of students included in the intervention studies examined in this study was 2531. Of the study designs, the most common was a quasi-experimental pretest/posttest study [18, 21, 24, 26, 29, 30, 35, 37, 40, 50], followed by the Mixed-method [22, 23, 36, 42, 46, 49], Quantitative survey-based study [19, 20, 27, 31, 34, 47], cross-sectional study [25], one-group posttest-only design [32, 44, 45], case study [38, 48], observational [41] quasi-experimental with post-test only [43], pretest/posttest [22], experimental design with qualitative and quantitative data collection [33], and design and development of a game-based language learning system [39]. A summarized data on the objectives, measurement tool, study design, outcomes, and challenges from reviewed articles are categorized in Table 4.

**Table 4:** Data of objectives, measurement tool, study design, outcomes, and challenges from reviewed articles

Authors	Objectives	Measurement tool	Study design	Outcomes	Challenges
Burgos et al. (2024) [18]	• Design an educational intervention using software and traditional indoor games to increase participants’ • motivation for learning new vocabulary in EFL	Observation Interviews Pretest/posttest	Interpretative Paradigm & Mixed-method	• Increased knowledge and motivation	Not-applicable
Zijun Shen et al(2024) [19]	• Investigate the influence of gamification integration on language learning achievement and motivation among Chinese students	Surveys	Mixed-method	• Highlighted the significance of personalized gamification approaches in enhancing language proficiency and the critical role of intrinsic motivation and learning preferences	Not-applicable
Huseinović L.(2023) [20]	• Investigate the impact of gamification on student motivation and academic performance in the context of TEFL	Questionnaire	Mixed-method	• Enhanced student motivation • Improved academic performance • Increased engagement in language learning	Not-applicable
EA Simbaña-Simbaña et al(2023) [21]	• Improve oral communication skills in EFL students via gamification	Observation Questionnaire	Quasi-experimental with pretest/posttest	• Improved students’ oral communication skills • Promoted active participation /motivation	Not-applicable
Önal, Nezih et al(2022) [22]	• Demonstrate the effectiveness of the mobile game in enhancing mobile learning tools acceptance, motivation in English, and mobile learning attitudes among students	Questionnaire	Quasi-experimental with pretest/posttest	• Enhanced motivation in learning English • Positive attitude development toward mobile learning	Not-applicable
Chan Sumie et al(2022) [23]	• Examine the teacher’s/students’ perceptions regarding the effectiveness of gamification in teaching/learning motivation in online environments	Questionnaire Interviews	Mixed-method	• Positive attitudes towards incorporating games in classrooms to enhance learning motivation.	• Some teachers disagreed with the effectiveness of gamification
Xiuhan Li et al(2022) [24]	• Investigate how gamification can facilitate self-regulation among students in e-learning EFL environments	Reading Tests Questionnaire Interviews	Quasi-experimental with pretest/posttest	• Enhanced self-regulation • Increased motivation and engagement in learning activities	• Difficulties with internet connectivity and access • Struggled to maintain motivation and engagement throughout the gamified learning process • Variability in students’ self-regulation skills affected their ability to effectively engage with the gamified elements.
Sorayyaei Azar et al(2020) [25]	• Explore the effectiveness and application of ICT technologies (mobile-assisted language learning, gamification, and VR in enhancing EFL during the COVID-19 pandemic	Questionnaire	Mixed-method	• Improved student engagement, motivation, and learning outcomes in EFL acquisition	• Technology access, student engagement levels, and the need for effective implementation strategies in diverse learning environments
Marina Purgina et al(2020)[26]	• Explore the effectiveness of gamification of natural EFL grammar learning through the WordBricks mobile app	Pretest/posttest	Quasi-experimental with pretest/posttest	• Improvement in grammar acquisition and engagement in language learning • Engages users in game-like behavior • Enhances learning through experimentation • Provides immediate feedback	• User interface limitations • Limited vocabulary • Inadequate user feedback
Wong Mee Mee et al(2020) [27]	• Explore pre-service teachers’ views on the role of gamification in classroom teaching	Questionnaire	Quantitative	• Improved learners’ interest in language learning subconsciously	• Some teachers found it challenging to use in the classroom Barriers faced by pre-service teachers in implementing gamified activities
YANES N et al [28]	• Explore the use of gamification and serious games for EFL	Questionnaire	Mixed-method	• Enhance the learning experience and motivation	Not-applicable
Lam, Hew, & Chiu (2018) [29]	• Investigate the effectiveness of a gamified blended learning approach on improving students’ argumentative writing skills and the impact of digital game mechanics on student engagement and writing performance	Written essay Interviews	Quasi-experimental with pretest/posttest	• Enhanced student engagement • Improved writing performance.	Not-applicable
Ling (2018) [30]	• Measure the impact of meaningful gamification on students’ motivation to read background material and grasp key concepts	Questionnaire	Mixed-method	• Enhanced motivation and engagement in reading tasks	• Students’ lack of motivation to read background material, their difficulty in accurately understanding key issues even when motivated, and the resulting negative impact on their seminar contributions and paper writing skills.
Castañeda, Guerra, & Ferro (2018) [31]	• Show the use of technological tools and their integration into the education system through gamification	Questionnaire	Mixed-method	• Enhanced learning outcomes and improved educational performance.	• Technical problems
Kirsi Korkealehto(2018) [32]	• Evaluate the effect of gamification on students’ success and involvement in a formative evaluation context	Posttest	Mixed-method	• Enhanced student engagement • Foster language learning • Offer positive learning experiences.	• The learning environment differed from the learner’s prior experiences with language learning
Won and Kim (2018) [33]	• Present a practical implementation of SNS-based self-directed English learning and evaluate its effectiveness	Surveys Interview Analysis of Facebook interactions	Mixed-method	• Enhanced engagement and improved learning outcomes.	Not-applicable
Sun and Hsieh (2018) [34]	• Investigate whether a gamified interactive response system can improve EFL learners’ motivation, engagement, and attention	Questionnaire	Quasi-experimental	• Improved motivation, engagement, and attention in EFL learners	Not-applicable
Homer R et al(2018) [35]	• Compare the effects of digital badges and points versus a non-digital classroom token system on student behavior and English learning	Pretest/posttest	Field-experiment/Quasi-experimental	• Improvement in classroom behavior and English learning • Enhanced student engagement • Positive behavioral changes observed in the digital badges group • Enjoyment reported by students using digital badges	• Limited effectiveness in younger grades (Grades 1 and 2 showed no significant improvement) • Specific limitations of the study duration and the generalizability of findings were not detailed.
Guaqueta and colleuges(2018) [36]	• Explore the effectiveness of language learning apps in enhancing vocabulary acquisition among EFL learners	Assessments Student feedback	Mixed-method	• Improved vocabulary recall, motivation, and engagement among students	• Need for ongoing support and additional practice for students struggling with vocabulary
Bustillo, J, et al(2017) [37]	• Examine how a Mobile Assisted Language Learning application influences learners’ attitudes towards language learning in mandatory and voluntary contexts	Pretest/posttest	Mixed-method	• Enhanced learning process, increased motivation, and willingness to continue using the application.	• Some participants noted a lack of human feedback and simplicity of use, which may affect the overall learning experience.
Mchucha, Ismaeil and Tibok (2017) [38]	• Explore whether a gamification-based interactive thesaurus app could improve English vocabulary among undergraduate students	Open-ended interview	Mixed-method	• Students preferred mobile learning over traditional approach and online platforms over mobile apps Increased knowledge • Increased motivation and enjoyment of learning	• Need for further investigation on effectiveness Lecturers should focus more on the explicit use of mobile technology in language learning
Zhou, Yu and Shi (2017) [39]	• Design and develop a game-based language learning system called ADVENTURE to improve learners’ skills and self-motivation for language learning	Focus group Survey	Mixed-method	• Enhanced learning experience and improved learning outcomes.	Not-applicable
Medina and Hurtado(2017) [40]	• Explore the use of the Kahoot online platform as a tool for teaching/learning vocabulary in EFL class	Survey	Quasi-experimental	• Improved engagement, interaction, motivation, and vocabulary acquisition	• Wireless connection • Limited study time
Girardelli, D.(2017) [41]	• Build students’ ability to communicate orally and foster their understanding of impromptu speaking through a gamified activity that embraces students’ use of texting and social media	Observation	practical application design	• Increased creative engagement • Reduced speech anxiety in impromptu speaking scenarios	• Small sample Short study time
Baldauf et al [42]	• Investigate the requirements and acceptance of a mobile gamified learning companion in a blended learning context for language teaching	Questionnaire/Interview	Mixed-method	• Enhanced engagement and acceptance among students, teachers, and parents.	Not-applicable
Hung H.T (2017) [43]	• Identify and categorize learners’ perceptions of using clickers in a BYOD model within a flipped classroom	Quiz/Post-intervention survey/Individual interviews	Quasi-experimental	• Enhanced engagement	• Technical complexities such as Wi-Fi infrastructure and internet speed were highlighted as potential limitations.
Kayımbaşıoğlu et al. (2016) [44]	• Explore the integration of gamification technology in educational settings and its effects on student engagement and learning outcomes	Questionnaire	Mixed-method	• Improved language acquisition and awareness of the peace concept. • Technology-assisted learning minimizes the distraction of children and boosts the learning curve	Challenges in effectively integrating gamification into existing curricula • Need for further research to understand the long-term impacts of gamification on student learning outcomes
Zarzycka-Piskorz (2016) [45]	• Observe/assess how gamification, (through the Kahoot platform) influences students’ motivation to learn/practice grammar	Questionnaire	Mixed-method	• Enhanced student motivation and engagement in grammar learning.	Not-applicable
Zhi Quan(2016) [46]	• Explore the effectiveness of mobile data-driven learning in enhancing vocabulary learning among academic English learners	Passive data capture Questionnaires Interviews	Quasi-experimntal	• mobile data-driven learning can facilitate vocabulary learning through contextual exposure to authentic language.	• The approach was not well accepted by intermediate-level students, indicating a need for major adjustments to make mobile data-driven learning more effective and acceptable.
Hasegawa et al(2015) [47]	• Develop a mobile application supporting sustainable motivation in learners for English vocabulary learning through gamification	Questionnaire	Mixed-method	• Improvement in learners’ motivation, engagement, and vocabulary acquisition. • Flexibility in learning	• Learner fatigue from gamified elements and challenges in maintaining engagement over time.
Lui(2014) [48]	• Explore how gamification can enhance vocabulary learning among EFL students	Labs games	Case-study	• Increased engagement and motivation in vocabulary learning.	Not-applicable
Abrams et al (2014) [49]	• Explore how gamification can help the development of vocabulary among adolescents/to assess the impact of adaptive online resources on vocabulary learning	Observation Interview	Mixed-method	• Improved understanding of vocabulary in context, Increased enjoyment of learning • Heightened awareness of personal word knowledge	Not-applicable
Sandberg et al(2011) [50]	• Investigate the added value of mobile technology for learning EFL among primary school students	Questionnaires	Quasi-experimental	• Enhanced vocabulary mastery, increased motivation to learn, and the ability to learn in informal contexts outside of school.	Not-applicable

### Modes of delivery

According to a study published by Ladyshewsky. R and colleagues [51], delivery modes are categorized into three types face-to-face, blended, and pure online. Blended delivery involves online and face-to-face learning, while pure online delivery involves only online learning. Of the 33 studies, 15 studies used pure online modes of delivery [18–25, 33, 35, 39, 45–47, 49], 11 used blended learning [29–32, 34, 36, 37, 40, 42, 43, 50], and 7 studies used face-to-face delivery mode [26–28, 38, 41, 44, 48].

### Technology used

Of the studies reviewed, 11 utilized Mobile learning [22, 25, 26, 37, 39, 42, 43, 45–47, 50], 9 utilized online learning platforms [19, 21, 24, 29, 32, 36, 38, 40, 41], 3 utilized digital games and gamification platforms [20, 27, 30], 2 utilized Information and communication technologies-web based environment [44, 48], one used simulation [32], one used Online adaptive resources [49], one used Interactive Response system [34], one used Facebook as a social networking service [33], one used augmented reality [31], and one used software games requiring facilitates of Internet connectivity [18].

### Learning environment

Of the reviewed studies, 8 studies utilized self-designed software, tailored to the needs of the study [23, 34, 39, 43, 44, 46, 47, 50], software games requiring facilitates of Internet connectivity [18, 25], online learning environment [19], various gamified educational tools [20, 27, 28, 32], Zoom [21], Tenses application [22], Oxford Achiever [24], WordBricks [26], Edmodo [29], Protégé [30], Caterpillar count [31], Facebook [33], ClassDojo [35], Duolingo [36, 37], gamification-based interactive thesaurus application [38], Kahoot [40, 45], Feelbot [41], Duolingo and Babbel [42], Jeopardy [48], Online adaptive resources [49].

### Gamification element concept/mechanism/feature

Various game elements were utilized in 33 reviewed studies, 4 studies not exactly mentioned the game elements [18, 20, 25, 27], challenges [19, 23, 24, 26, 30, 34, 36–40, 42, 48, 49], badges [35–37, 40, 44, 49], leader board [24, 29, 31, 32, 35, 36, 40, 42–45, 47], points [22, 24, 25, 27, 29, 35–38, 40, 43, 47, 48], and leveling [19, 23, 30, 31, 36, 37, 39, 44, 45, 47], feedback [19, 21, 22, 26, 29, 30, 33–43, 45–50], time [22, 26, 34, 40, 43, 47, 48, 50], story/narration [22, 30, 39, 41], progress bar [26, 36, 37, 39, 49], Badge [21, 30, 37, 39, 40, 48, 49], avatar/character [30, 35, 48], Competition [39]. (Table 5)

**Table 5:** Frequency of gamification elements

Gamification element	Frequency	Gamification element	Frequency
Feedback	23	Badge	7
Challenge	14	Progress bar	5
Points	13	Story/narration	4
Leaderboard	12	Digital badges	3
Leveling	10	Avatar/character	3
Time	8	Competition	1

### Measurement tool

Of measurement tools, the most frequently utilized tool was Questionnaires(*N* = 20) [19–25, 27, 30–32, 34, 35, 37, 42, 43, 45, 47, 49, 50], and content analysis(*N* = 8) [22, 29, 33, 38, 42, 43, 46, 49], pretest/posttest(*N* = 6) [21, 26, 29, 30, 36, 40], observation [21, 41], survey [21, 33], analysis of interactions [33], focus group [39], review [23], data collection [46].

### What was analyzed

Of reviewed studies, 15 analyzed Knowledge [18, 20–22, 26, 29–31, 33, 37, 39, 40, 46, 47, 50], 6 analyzed attitude [20, 22, 37, 42, 46, 49], 18 analyzed motivation [18, 20–22, 29–31, 33, 34, 37–40, 43, 45, 47, 49, 50], 7 analyzed engagement [26, 29, 30, 33–35, 39], 2 analyzed skill [29, 31], and others analyzed speaking skills [41], reducing anxiety [41] behavior [35], satisfaction [33, 43], and perceived improvements [20].

### Content of language learning

Of 33 reviewed studies, 10 studies analyzed vocabulary [18, 21, 34, 36, 38–41, 47, 49], 5 analyzed writing [20, 29, 31, 33, 42], 5 analyzed speaking [20, 31, 33, 35, 42], 5 analyzed reading [20, 30, 33, 35, 39], 5 analyzed grammar [21, 26, 39, 42, 45], 4 analyzed listening [31, 33, 37, 42], 3 analyzed pronunciation [21, 39, 42], and one analyzed perception [20].

### Outcomes of using gamification

As shown in Table 4, all included papers reported some outcomes. Increase in the motivation was the most frequently reported outcome (21 articles) [18–25, 27, 30, 33, 34, 36–40, 45, 47, 49, 50], followed by knowledge (14 articles) [18–21, 26, 29, 31–33, 35, 40, 44, 46, 50], (*n* = 12) Improved user engagement [24–26, 29, 30, 32–34, 36, 37, 39, 40, 42–45, 47], attitude (*n* = 7) [22–24, 27, 42, 46, 49], increased enjoyment (*n* = 2) [38, 41], improved learning skills [29, 39]. Other studies reported increased flexibility [47], behavior [35], attention [34], self-regulation [24], performance [31], participation [21], speaking skills [41], learning experience [43], vocabulary learning [46], and one reported minimized distraction and boosts the learning curve [44].

### Challenges in Implementing Language Gamification in EFL classrooms

Reviewed studies found that the integration of gamification in EFL classrooms presents several challenges that educators must navigate to ensure effective implementation. These challenges can significantly impact both teaching practices and student learning outcomes. Educators often struggle with incorporating gamification elements such as feedback, points, and rewards into their teaching methods. Effective implementation strategies are crucial, as many teachers report difficulties applying gamified classroom activities in the classroom [27]. Furthermore, the learning environment may differ significantly from students’ previous experiences with language learning, complicating the transition to gamified approaches [32]. Access to technology is a significant challenge, as disparities in technology availability can hinder the effective use of gamification [24, 25]. Additionally, issues such as internet connectivity [40, 43] and the limitations of mobile devices—such as small screens and restricted input mechanisms—and technical problems can restrict the type of content that can be effectively delivered [31, 50]. These technological constraints necessitate careful design considerations to ensure that learning materials are accessible and engaging.

Maintaining student engagement over time is another critical issue. Studies indicated that while gamified elements can initially boost motivation, they may also lead to learner fatigue if not managed properly [47]. Moreover, varying levels of student engagement can affect the overall efficacy of gamified learning environments [25]. Integrating gamification into existing curricula poses additional challenges. Educators must align gamification elements with educational objectives to prevent negative impacts on learning outcomes [24]. The need for further research into the long-term effects of gamification on student learning outcomes is also highlighted [44].

Professional development for educators is essential to equip them with the skills necessary for implementing gamified learning effectively [22]. Many teachers may lack experience or confidence in using gamification techniques, which can impede successful integration into their teaching practices.

User interface limitations can also hinder the effectiveness of gamified applications. Inadequate user feedback and limited vocabulary options can detract from the learning experience [26]. Additionally, some participants have noted that the simplicity of use and lack of human feedback may negatively affect engagement and learning outcomes [37]. Other challenges that can be mentioned consist of the need for effective implementation strategies in diverse learning environments [23, 25] implementation time limit [40], and a need for major adjustments to make mobile DDL more effective and acceptable [46].

## Discussion

This scoping review analyzed 33 studies on gamified applications in TEFL, revealing important trends and insights in the field. The majority of publications were from 2018, with a significant concentration in Asia, indicating a growing interest in gamification in education. The studies predominantly utilized quasi-experimental pretest/posttest designs, suggesting a preference for methodologies that facilitate measurable comparisons of gamification interventions. Online delivery was the most common instructional mode, reflecting the increasing use of digital platforms in language education. Mobile learning technologies were frequently employed, demonstrating their effectiveness in creating engaging and accessible gamified experiences. Many studies also utilized self-designed software tailored to specific learning objectives.

Since 2018, there has been a noticeable decline in the number of articles on this topic. While it is possible that relevant studies exist but were not included in our search, a likely reason for this reduction is the evolving landscape of educational technologies, particularly the rise of Artificial Intelligence (AI) and various interactive tools. Although early forms of AI, such as GPT-1, were introduced in 2018, their impact on language education became more pronounced when models like GPT-3 arrived in 2020, which enhanced personalized learning experiences and engagement strategies [52]. Moreover, interactive tools have been a part of language education for a long time, their sophistication and integration into curricula have increased dramatically over the last few years. This evolution has been accompanied by a shift in focus from gamification alone to a broader array of technology-enhanced learning methods [53]. It is worth noting that concepts like gamification often experience cycles of enthusiasm followed by periods of diminished interest, similar to other technology-related trends in language learning since the early 1950s. As educators explore new methodologies, gamification may have receded from the forefront as other innovative approaches gain traction [54].

However, even though we could deduce some of the important implications not directly stated in the literature available to us from our analysis, we realize that such inferences must be presented cautiously. The history of educational technology will suggest that any claims for one method replacing another must be made guardedly and supported by empirical evidence.

Among utilized elements, feedback was the most commonly used among the gamification elements, emphasizing its importance in enhancing student motivation and engagement, followed by challenges that foster a competitive spirit among learners. Immediate feedback is a pivotal component of gamification that significantly enhances the learning experience across various educational contexts [55], including language learning and medical education [56]. This type of feedback refers to the timely and specific responses provided to learners after they complete a task or make a decision [57]. It plays a crucial role in motivating learners, helping them quickly identify mistakes, and allowing for real-time adjustments to their strategies [58]. In traditional educational settings, students often have to wait for assessments or assignments to receive feedback, which can hinder their ability to improve and progress effectively [59]. However, gamification addresses this limitation by integrating immediate feedback mechanisms into learning [60]. Incorporating immediate feedback in gamified learning environments fosters a cycle of continuous improvement [55]. For instance, when learners engage with quizzes or challenges that provide instant results, they can promptly recognize their strengths and areas needing improvement [61]. This real-time information is essential for maintaining motivation, as it reinforces a sense of accomplishment when learners succeed and encourages them to strive for better performance when they encounter difficulties [62]. Studies have shown that immediate feedback not only enhances engagement but also increases retention of information by promoting frequent practice and reinforcing learning through visual cues such as leaderboards [63], badges [64], points [65], and progress bars [66]. Moreover, immediate feedback contributes to personalized learning experiences [67]. By utilizing data collected during gamified activities, educators can tailor feedback to individual learner needs, ensuring that each student receives targeted guidance that aligns with their unique learning paths [68]. This adaptive approach not only maximizes learning potential but also empowers learners to take ownership of their educational journey [69].

Furthermore, different gamification elements—such as immediate feedback and competitive features—align uniquely with the demands of each field. In medical education, immediate feedback is critical for reinforcing knowledge retention during high-stakes training scenarios [70]. In TEFL, while immediate feedback also plays a role in enhancing learning outcomes, the emphasis may be more on fostering communication skills through interactive dialogues rather than competition.

The findings from the scoping review revealed that increased motivation was the most commonly reported outcome of gamified applications in TEFL. This result can be attributed to several factors. First, gamification integrates game elements such as points, badges, and challenges, which make learning interactive and increase student engagement, making education enjoyable. According to research, these elements enhance the sense of achievement and competition among students, increasing their intrinsic motivation significantly [19, 34]. This outcome closely mirrors findings in medical education, where gamification strategies have also been shown to significantly enhance student engagement and participation [71, 72].In both cases, gamification transforms traditional educational experiences into dynamic and immersive ones [73, 74]. However, while gamification is effective in enhancing motivation, it may not be the magic bullet. The effectiveness of gamification may depend on several factors including specific educational contexts, student demographics, and individual learning preferences. Gamification transforms traditional educational experiences in language education, as well as in other disciplines, into dynamic and immersive ones [2]. However, claims about the effectiveness of gamification should be approached with caution. As noted, concepts like gamification often experience cycles of enthusiasm followed by periods of diminished interest. This cyclical nature reflects broader trends in educational technology, where initial excitement may wane as new methodologies emerge [75].

The comparative discussion between TEFL and medical education offers valuable insights into the implementation of gamification. Both fields report increased motivation and engagement as significant outcomes; however, they face distinct challenges. For instance, medical education often employs virtual patient simulations that allow students to apply theoretical knowledge in practical settings, reinforcing their learning while enhancing motivation simultaneously [76]. In contrast, language educators may struggle with integrating similar immersive experiences due to resource constraints or curriculum rigidity.

In medical education, gamified platforms often feature virtual patient scenarios and interactive simulations that enable students to apply theoretical knowledge in practical settings, reinforcing their learning and enhancing motivation simultaneously [56, 77]. Additionally, the competitive elements of gamification in education—such as leaderboards and challenges—appeal to students’ innate desires for achievement and recognition, nurturing a sense of accomplishment that encourages ongoing participation [63, 78]. This is especially important in medical education, where the stakes are high and students are eager to excel in their training [79, 80].

As this study revealed, gamified learning experiences also improved knowledge retention by allowing students to apply theoretical concepts in practical scenarios. In medical education, interactive simulations and quizzes help reinforce learning outcomes through immersion [81]. The pattern that also emerges reinforces the assertion of gamification being sound pedagogically because motivation increases in both language learning and medical education. By encouraging learners to be more active and engage in the educational process by using interactive gamification elements, gamification gives relevance to their education and better prepare individuals for future challenges both within and outside of their fields of study.

This study showed that gamified learning experiences can enhance knowledge retention by enabling students to apply theoretical concepts in real-world situations. In the field of medical education, interactive simulations and quizzes play a crucial role in reinforcing learning outcomes through immersive experiences [82]. Although increased motivation is a key advantage of gamification, it’s important to understand that motivation by itself doesn’t guarantee that a technique is pedagogically effective. The effectiveness of instructional strategies in achieving desired learning outcomes is what defines pedagogical soundness. While there is a link between gamification and heightened motivation that can boost student engagement, this connection should be approached with care. It’s essential to assess the actual impact of gamification on learning effectiveness through empirical research. By encouraging learners to take a more active role in their education through interactive gamification elements, educators can design relevant and engaging learning experiences that better equip individuals for future challenges, both in their fields and beyond. Therefore, while gamification has the potential to improve motivation and engagement, its true effectiveness should be evaluated based on measurable learning outcomes.

The implementation of gamified learning presents several challenges that need to be addressed to maximize its effectiveness in educational settings. Key issues include learner fatigue, technological access disparities, and the integration of gamification into existing curricula. To mitigate learner fatigue, educators can carefully pace gamified activities and ensure they are varied to maintain student interest. For instance, incorporating a mix of game elements—such as quizzes, interactive simulations, and collaborative challenges—can help sustain engagement over time. Additionally, providing students with opportunities for self-directed learning within the gamified framework allows them to take breaks and choose activities that align with their interests and energy levels. Addressing technological access disparities is crucial for equitable implementation of gamified learning. Educators can provide alternative resources or training for students who may lack access to advanced technology. For example, offering offline versions of gamified activities or utilizing low-tech solutions can help bridge the gap for students without reliable internet access or modern devices. Furthermore, schools can explore partnerships with local organizations to provide necessary technology or internet access to underserved populations. Integrating gamification into existing curricula can be challenging due to rigid educational structures. To facilitate this integration, educators should seek to align gamified activities with established learning objectives and standards. Collaborating with colleagues across disciplines can also foster a more cohesive approach to incorporating gamification into the curriculum. Sharing successful case studies from other educational contexts can provide valuable insights and inspire innovative strategies for overcoming integration challenges.

### Research gap

Finally, there is a pressing need for further investigation into the effectiveness of gamification across different educational contexts [38]. Specific limitations related to study duration and generalizability of findings have been noted, particularly in younger grades where no significant improvement was observed [35]. This underscores the necessity for ongoing research to better understand how gamification can be optimized for diverse learner populations. Also, Future research directions can focus on integrating gamification with AI and other emergent technologies to assess their combined impact on learning outcomes. For instance, studies could explore how AI can enhance gamified learning experiences by providing real-time feedback tailored to individual learners’ needs or by personalizing game elements based on student performance data.

## Conclusion

This scoping review has discussed the contributions of gamified applications to TEFL and the identification of key trends and effective practice outcomes that characterize it. It is quite important to note that the review emphasizes how this gamification enhances student motivation, increases engagement, and takes the learning process to active learning through interactive elements, marking a transformative shift in educational practices. The review highlights that gamified applications can transform traditional educational experiences into dynamic and immersive ones, fostering active learning.

Long-term studies of gamification and its differential effects among different groups of learners and emergent technologies are some aspects that need investigation in the future within the gamified learning environment. While our findings indicate that gamification can improve student performance, it is crucial to recognize its broader implications for preparing learners to face various life challenges. By integrating gamified learning experiences in language education, students develop critical skills such as problem-solving, collaboration, and resilience—qualities essential for navigating real-world situations. For instance, gamified activities often simulate real-life scenarios that require students to think critically and adapt their strategies, thereby equipping them with the tools necessary to tackle future challenges. However, it is also important to acknowledge the potential challenges associated with implementing gamification in educational settings. Issues such as learner fatigue, technological access disparities, and curriculum integration can hinder the effectiveness of gamified approaches. Most of these challenges can be effectively addressed through strategic planning and careful attention to implementation practices. By proactively considering these issues, educators can maximize the positive impact of gamification on their students’ learning experiences.

## Data Availability

Yes, the data analyzed during the current study are available from the corresponding author on reasonable request.

## Abbreviations

TEFL: Teaching English as a foreign language
VR: Virtual Reality
EFL: English as Foreign Language
